# Trends and disparities in anencephaly-related early childhood mortality in the United States between 1999 and 2020: A nationwide CDC WONDER analysis

**DOI:** 10.1097/MD.0000000000046593

**Published:** 2025-12-26

**Authors:** Hashim Mohamed Siraj, Akshaya Srinivasan, Akshit Valsaraj Chemmancheri, Samiksha Ravindra Balaguragi, Vaishnavi Suresh, Muhammad Husnain Ahmad, Masab Ali, Angeline Abraham, Anushka Sen, Ajin Mathews John, Bilal Muhammad, Nupura Ajesh, Nikhila Liz Aby, Muhammad Ibrahim, Insha Samsulhuda Khan, Courtney Truebody, Nirjhar Yaduvanshi, Udgam Yaduvanshi, Mohammed Alsabri

**Affiliations:** a Ivane Javakhishvili Tbilisi State University, Tbilisi, GA; b Tbilisi State Medical University, Tbilisi, GA; c Ivane Javakhishvili Tbilisi State University Faculty of Medicine, Tbilisi, GA; d S. Tentishev Asian Medical Institute, Kyrgyzstan; e Punjab Medical College, Faisalabad, Pakistan; f Georgian National University SEU, Tbilisi, GA; g Georgian American University, Tbilisi, GA; h New Vision University School of Medicine, Tbilisi, GA; i Alte University, Tbilisi, GA; j Saint Christopher’s Hospital for Children, Philadelphia.

**Keywords:** anencephaly, early childhood, folic acid, mortality, NTDs

## Abstract

Anencephaly is a severe neural tube defect that affects approximately 1 in every 5250 live births in the United States. This fatal congenital condition is linked to modifiable risk factors, including folic acid deficiency, maternal diabetes, and exposure to teratogenic medications such as certain anti-epileptics. Despite public health efforts to promote folic acid supplementation, disparities in mortality persist. This study aimed to evaluate national trends in anencephaly-related early childhood mortality from 1999 to 2020 using the CDC Wide-Ranging Online Data for Epidemiologic Research (CDC WONDER) database. We analyzed death certificate data from the CDC WONDER system to assess trends in anencephaly-related mortality among children from birth to 4 years of age between 1999 and 2020. Age-adjusted mortality rates (AAMRs) per 100,000 population and annual percent changes (APCs) with 95% confidence intervals (CIs) were calculated. Mortality trends were stratified by sex, race/ethnicity, U.S. Census region, state, and 2013 urbanization classification. Between 1999 and 2020, there were 6969 anencephaly-related early childhood deaths in the United States, with 89.7% occurring in medical facilities. The overall AAMR declined from 1.7 in 1999 to 1.5 in 2020 (APC: −0.43; 95% CI: −0.94 to 0.09), with peaks in 2000 and 2012 (AAMR: 1.8). Females consistently had higher AAMRs than males (1.8 vs 1.4). By race, Hispanic individuals had the highest AAMR (2.0), followed by NH White (1.7) and NH Black (1.2), with significant declines among Hispanic and NH Black population. Regionally, the highest AAMR was in the Midwest (1.9), and the lowest in the Northeast (1.0). Among states, South Dakota had the highest (3.2), and Connecticut the lowest (0.5). Noncore metropolitan areas exhibited the highest AAMR (2.1), with varying temporal trends across urbanization levels. Anencephaly-related early childhood mortality has declined in the United States from 1999 to 2020; however, disparities remain. Higher mortality rates persist among Hispanic/Latino populations and in nonmetropolitan areas, highlighting the need for targeted public health interventions. These findings underscore the importance of expanding access to folic acid supplementation, culturally tailored maternal health education, and equitable prenatal care services, especially in underserved communities.

## 1. Introduction

Anencephaly is a severe neural tube defect resulting from the failure of neural tube closure during early embryogenesis, typically the third to fourth week of development.^[1]^ This results in the absence of the fetus’s brain, skull, and scalp, which makes survival after birth unlikely. The global incidence of anencephaly is 8.3 per 10,000 births, while the mortality rate is 5.5 per 10,000 births.^[2]^ This congenital anomaly is associated with environmental factors like folic acid deficiency, maternal health conditions like diabetes, and the usage of teratogenic medications like valproic acid and carbamazepine.^[3]^ Following the FDA’s mandate in 1996 to fortify enriched cereal grain products with folic acid, a significant decline in neural tube defects (NTDs) has been observed. Namely, between 1996 and 2001, the prevalence of anencephaly decreased by 21%. This reduction translates to about 920 fewer infants being born with NTDs every year.^[4,5]^

Despite public health interventions such as prenatal and maternal screening using ultrasound and maternal serum alpha-fetoprotein (MSAFP) testing, ethical and emotional disparities continue to exist. Therefore, given its devastating prognosis, understanding the mortality rate for anencephaly can improve prenatal care while addressing public interventions. This study aims to analyze the early childhood mortality trends in anencephaly in the United States from 1999 until 2020 by utilizing CDC WONDER (Centers for Disease Control and Prevention Wide-Ranging Online Data for Epidemiologic Research). By examining the changes in mortality, regional patterns, and demographic disparities, this research explores the effectiveness of the existing preventive patterns and suggests areas for further improvement.

## 2. Methods

### 2.1. Study setting and population

In this descriptive study, death certificate data were retrieved from the CDC WONDER (Centers for Disease Control and Prevention Wide-Ranging Online Data for Epidemiologic Research) database and examined from 1999 to 2020 to assess anencephaly-related mortality trends among the early childhood U.S. population. Individuals belonging to early childhood were categorized into 2 age groups: <1 year and 1 to 4 years. Case assessment was based on the presence of ICD-10 code Q00.0 in mortality records, representing the standardized classification for anencephaly within the congenital malformations of nervous system category (Q00-Q07). The same ICD code has been previously used to identify anencephaly in administrative databases.^[6]^ This dataset includes cause-of-death information from death certificates across all 50 states and the District of Columbia. It has been used previously to analyze mortality trends related to congenital anomalies. For case determination, we included all mortality records where anencephaly (Q00.0) was listed in either the primary cause-of-death field or any of the contributing cause fields. Early childhood was defined as children <1 year and 1 to 4 years of age, a cutoff used by previous studies.^[7]^ This secondary analysis of completely de-identified national mortality data did not require Institutional Review Board approval. It was conducted in accordance with STROBE (Strengthening the Reporting of Observational Studies in Epidemiology) guidelines for reporting.^[8]^

### 2.2. Data abstraction

Data were abstracted for year of death, population size, sex, race/ethnicity, location of death, state, U.S. census region, and urbanization level according to the 2013 National Center for Health Statistics Urban-Rural Classification Scheme.^[9]^ Urbanization levels were categorized as large central metropolitan, large fringe metropolitan, medium metropolitan, small metropolitan areas, micropolitan, and noncore nonmetropolitan areas. Race/ethnicity was classified as non-Hispanic (NH) White, NH Black or African American, Hispanic or Latino, NH American Indian or Alaska Native, and NH Asian or Pacific Islander. Regional classification followed U.S. Census Bureau definitions for Northeast, Midwest, South, and West.^[9]^

### 2.3. Statistical analysis

Crude and age-adjusted mortality rates (AAMRs) per 100,000 population were calculated annually from 1999 to 2020, stratified by sex, race/ethnicity, age group, urbanization level, state, and Census region, with 95% confidence intervals (CIs). AAMRs were standardized to the year 2000 U.S. population.^[9]^ Trends in anencephaly-related mortality were assessed using the Joinpoint Regression Program (version 4.9.0.0; National Cancer Institute) to calculate annual percent change (APC) and 95% CIs.^[10]^ To characterize temporal trends and identify significant changes in mortality rates, we implemented log-linear regression models. The threshold for statistical significance was maintained at *P* < .05 (two-tailed) to minimize Type 1 error probability.

## 3. Results

A total of 6969 deaths were accounted for in anencephaly-related early childhood deaths in the U.S. (aged < 1; 1–4 years) between 1999 and 2020 (Table S1, Supplemental Digital Content, https://links.lww.com/MD/Q973). Of these, 89.7% occurred within medical facilities, 0.3% at nursing home/long-term care facilities, 0.6% at hospices, and 7.8% occurred at home (Table S2, Supplemental Digital Content, https://links.lww.com/MD/Q973.

### 3.1. Annual trends for anencephaly-related AAMR

**Figure 1.**
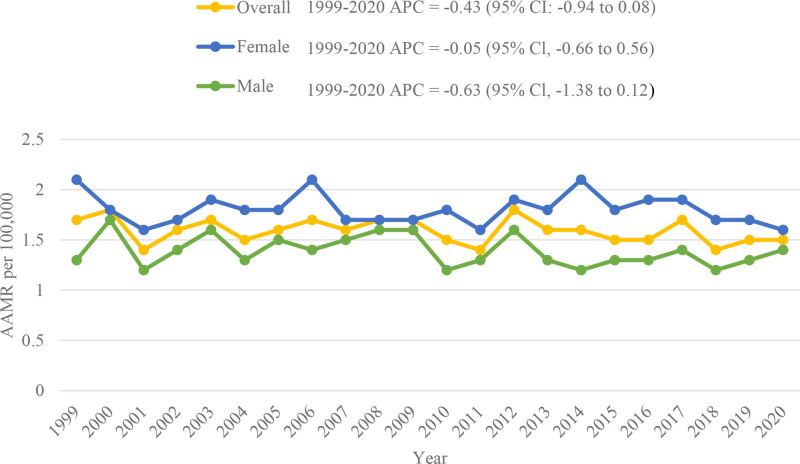
Overall and sex-stratified anencephaly-related AAMRs per 100,000 in early childhood in the United States, 1999 to 2020. AAMRs = age-adjusted mortality rates.

The AAMR for anencephaly-related deaths decreased from 1.7 in 1999 to 1.5 in 2020 (APC: −0.43; 95% CI: −0.94 to 0.09). The highest recorded AAMRs were observed in 2000 and 2012, both at 1.8 (Fig. 1, Tables S3 and S4, Supplemental Digital Content, https://links.lww.com/MD/Q973).

### 3.2. Anencephaly-related AAMR stratified by sex

Stratification by sex demonstrated consistently higher AAMRs in females than males throughout the study period (overall AAMR men: 1.4; women: 1.8). In males, the AAMR steadily increased from 1.3 in 1999 to 1.6 in 2008 (APC: 0.30; 95% CI: −2.55 to 3.23), followed by a decline to 1.4 in 2020 (APC: −1.27; 95% CI: −3.17 to 0.67). In females, the AAMR remained relatively stable from 1999 to 2017 (APC: 0.31; 95% CI: −0.49 to 1.11), followed by a steep decline from 2017 to 2020 (APC: −5.42; 95% CI: −16.93 to 7.68) (Fig. 1, Tables S3 and S4, Supplemental Digital Content, https://links.lww.com/MD/Q973).

### 3.3. Anencephaly-related AAMR stratified by race

**Figure 2.**
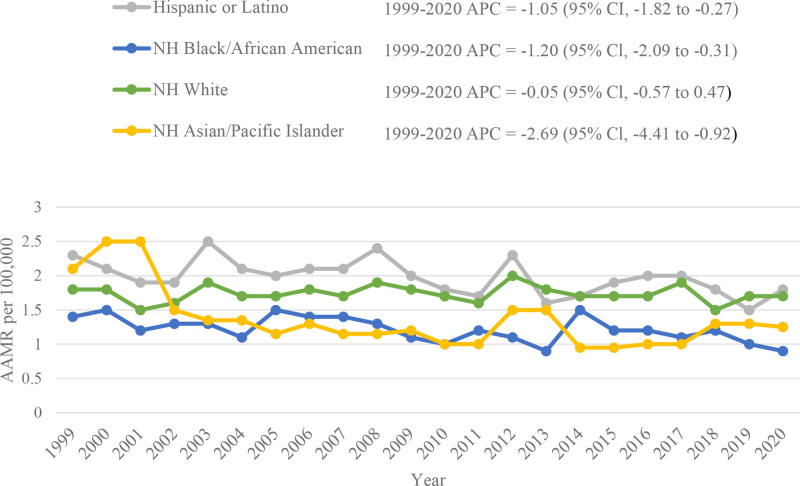
Anencephaly-related AAMRs per 100,000 stratified by race in early childhood in the United States, 1999 to 2020. AAMRs = age-adjusted mortality rates.

Stratification by race revealed that the overall AAMRs were highest among Hispanic or Latino individuals (2.0; 95% CI: 1.9–2.1), followed by NH White (1.7; 95% CI: 1.7–1.8), NH Black or African American (1.2; 95% CI: 1.1–1.3), NH American Indian or Alaska Native (1.1; 95% CI: 0.9–1.3), and NH Asian or Pacific Islander (1.0; 95% CI: 0.9–1.1). NH Asian or Pacific Islander populations demonstrated a significant reduction in AAMR between 1999 and 2005 (APC: −10.87; 95% CI: −19.95 to −0.74), followed by a period of stability until 2020 (APC: −0.06; 95% CI: −2.52 to 2.45). Both Hispanic (APC: −1.05; 95% CI: −1.82 to −0.27) and NH Black or African American (APC: −1.20; 95% CI: −2.08 to −0.31) populations demonstrated a comparable reduction in mortality between 1999 and 2020. The AAMR for the NH White population remained relatively stable throughout the duration of the study (APC: −0.05; 95% CI: −0.57 to 0.47) (Fig. 2, Tables S3 and S5, Supplemental Digital Content, https://links.lww.com/MD/Q973).

### 3.4. Anencephaly-related AAMR stratified by geographic region

**Figure 3.**
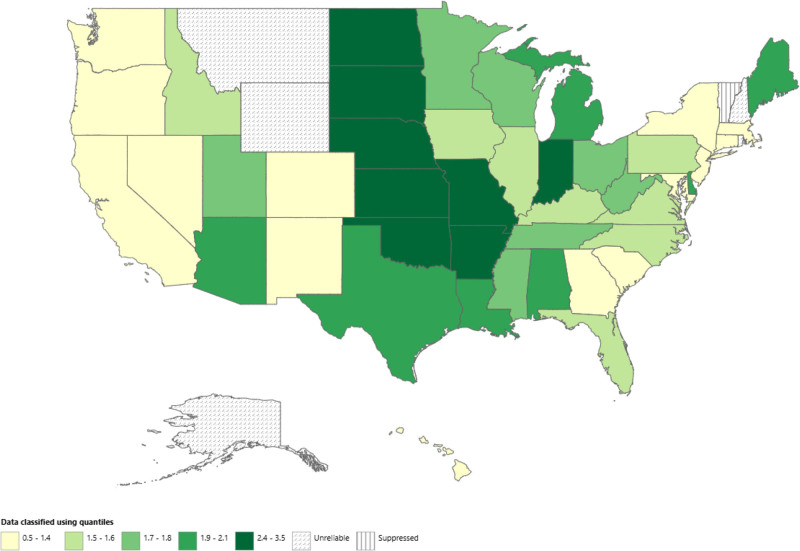
Anencephaly-related AAMRs per 100,000 stratified by state in early childhood in the United States, 1999 to 2020. AAMRs = age-adjusted mortality rates.

**Figure 4.**
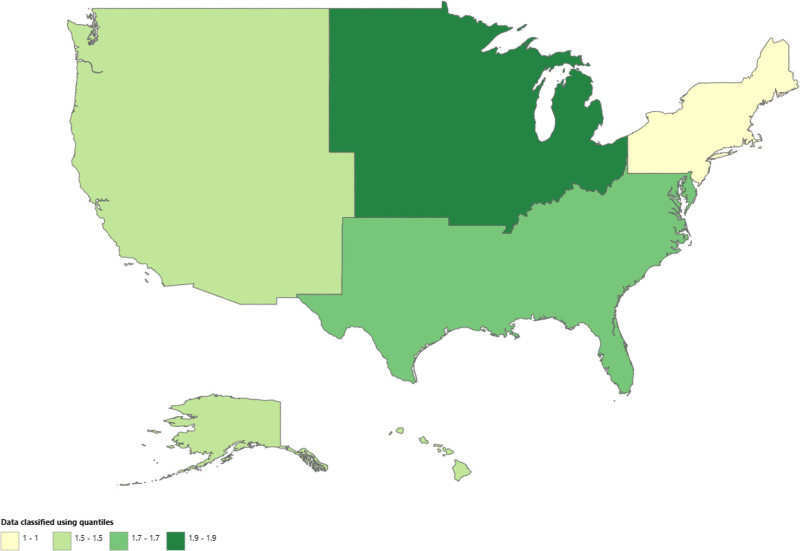
Anencephaly-related AAMRs per 100,000 stratified by census region in early childhood in the United States, 1999 to 2020. AAMRs = age-adjusted mortality rates.

The lowest anencephaly-related AAMR was observed in Connecticut at 0.5 (95% CI: 0.3–0.7) and the highest in South Dakota at 3.2 (95% CI: 2.3–4.4). States that fell in the top 90th percentile with the highest AAMRs were South Dakota (0.5; 95% CI: 0.3–0.7), North Dakota (3.0; 95% CI: 2.0–4.3), Oklahoma (2.4; 95% CI: 2.0–2.9) and Arkansas (2.4; 95% CI: 1.9–2.9). The states that were noted in the 10th percentile were Connecticut (0.5; 95% CI: 0.3–0.7), Massachusetts (0.7; 95% CI: 0.6–1.0), New York (0.7; 95% CI: 0.7–0.9), and New Jersey (0.8; 95% CI: 0.6–0.9) (Fig. 3, Table S6, Supplemental Digital Content, https://links.lww.com/MD/Q973). During the study period, the highest AAMR was observed in the Midwestern Region at (1.9; 95% CI: 1.8–2.0), followed by Southern (1.8; 95% CI: 1.7–1.8), Western (1.5; 95% CI: 1.4–1.5) and the least in Northeastern (1.0; 95% CI: 0.9–1.1) (Fig. 4, Table S7, Supplemental Digital Content, https://links.lww.com/MD/Q973).

### 3.5. Anencephaly-related AAMR stratified by urbanization

Overall, Noncore nonmetropolitan areas demonstrated the highest overall AAMR (2.1; 95% CI: 1.9–2.3). In metropolitan areas, large central metropolitan populations demonstrated a steady decline in AAMR from 1.7 in 1999 to 1.5 in 2017 (APC: −0.48; 95% CI: −1.26 to 0.30), followed by a steep decline to 1.2 in 2020 (APC: −6.24; 95% CI: −17.34 to 6.35). Large fringe metropolitan areas demonstrated an increase in AAMR from 1.3 in 1999 to 1.6 in 2015 (APC: 1.25; 95% CI: −0.57 to 3.10), followed by a decline to 1.1 in 2020 (APC: −8.95; 95% CI: −19.97 to 3.58). Medium metropolitan areas showed an initial increase in AAMR, from 1.7 in 1999 to 2.0 in 2002 (APC: 3.80; 95% CI: −11.70 to 22.04), followed by a steady decline to 1.7 in 2020 (APC: −0.37; 95% CI: −1.25 to 0.52). Small metropolitan areas demonstrated a steady decline in AAMR, from 2.6 in 1999 to 2.2 in 2020 (APC: −0.91; 95% CI: −2.09 to 0.28).

**Figure 5.**
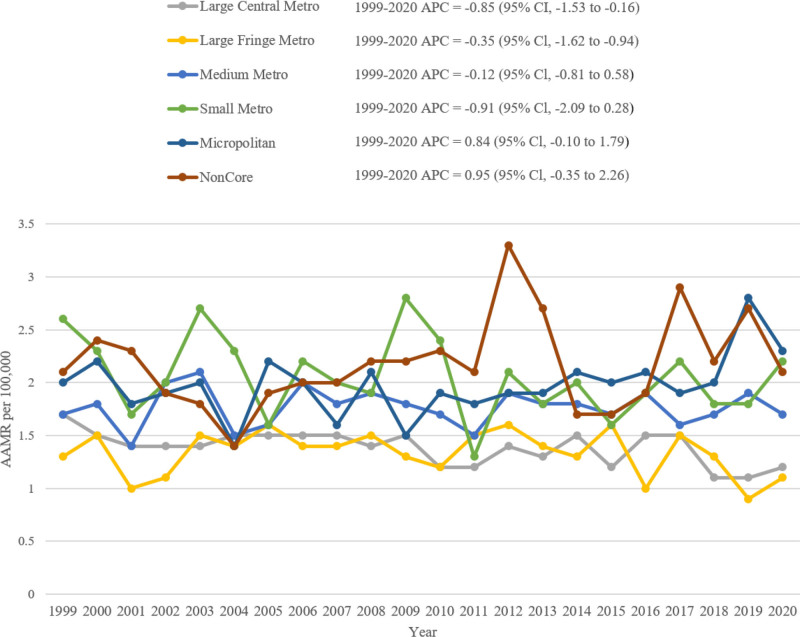
Anencephaly-related AAMRs per 100,000 stratified by urban–rural status in early childhood in the United States, 1999 to 2020. AAMRs = age-adjusted mortality rates.

In Micropolitan nonmetropolitan areas, the AAMR declined from 2.0 in 1999 to 1.8 in 2011 (APC: −0.85; 95% CI:−3.05 to 1.39), followed by a significant increase to 2.3 in 2020 (APC: 3.33*; 95% CI: 0.02–6.75). In Noncore nonmetropolitan areas, the AAMR declined from 2.1 in 1999 to 1.8 in 2003 (APC: −4.93; 95% CI: −20.59 to 13.80), followed by an increase to 2.1 in 2020 (APC: 1.71; 95% CI: −0.29 to 3.77) (Fig. 5, Table S8, Supplemental Digital Content, https://links.lww.com/MD/Q973).

## 4. Discussion

Between the years 1999 and 2020, from the Centers for Disease Control and Prevention, we identified specific trends that were critical in anencephaly-related early childhood mortality (Central Illustration, Supplemental Digital Content, https://links.lww.com/MD/Q973). Firstly, mortality rates declined significantly among Hispanic/Latino and Black/African American individuals, even though disparities persisted. Secondly, gender-based differences were noticed, with females initially facing a higher mortality rate than males, which gradually reduced by 2020. Lastly, geographical discrepancies emerged, with non-metropolitan areas displaying higher age-adjusted mortality rates when compared to metropolitan areas. These findings exhibit the need for focused interventions such as better prenatal care and folic acid supplementation in high-risk populations.

Anencephaly is a severe neural tube defect that has consistent outcomes of death; the trends and differences can be compared meaningfully with other causes of infant mortality, namely preterm birth, congenital anomalies, or even cardiovascular malformations.^[11]^ Fatality due to anencephaly is usually sudden but can occur shortly after infant birth due to the incomplete formation of the brain and the skull when compared to chronic or prolonged conditions such as congenital heart disease.^[12]^ In contrast to other leading causes, its distribution shows broader disparities in nutrition and access to healthcare in the prenatal phase. A significant fall in these neural tube defects, especially in anencephaly, is seen in the U.S. due to post-mandate fortification of folic acid,^[11–13]^ even though these advantages have not been shared equally over the geographic and racial groups.^[14]^ Constantly high rates in the non-Hispanic Black race and the Southern U.S., shown in the national infant mortality statistics, highly suggest that social factors of health, like education, poverty, and access to prenatal services, could add to both anencephaly-related as well as all-cause infant mortality discrepancies.^[14,15]^

The perseverance in the regional differences related to anencephaly mortality shows broader trends, especially in non-metropolitan and southern U.S., with these areas having increased all-cause childhood mortality rates. As mentioned by the CDC in the data from 2013, these regions face obstacles such as lower rates of utilization of maternal healthcare, insufficient access to specialist care, and increased socioeconomic disadvantage, which often, in turn, worsen the health disparities.^[15]^ Moreover, regardless of the decades of research showing the advantage of folic acid in preventing neural tube defects, voids in implementing the policies and coverage of the fortification continue to put specific populations at a higher risk.^[16]^ These findings have significant real-world implications: clinicians can identify high-risk groups earlier, public health policy can prioritize coverage expansion for supplementation programs, and food regulatory bodies can consider fortifying culturally relevant foods such as corn masa flour.^[16]^ Targeted efforts like these can help reduce preventable cases of anencephaly and close the disparity gap. Around the globe, the absence of mandatory folic acid fortification in some regions results in a certainly avoidable strain of anencephaly and spina bifida, fortifying the need for unified international policies and action along with it.^[17]^ Access to healthcare appears to be unequal in the U.S., with Medicaid expansion having an important role in better maternal and early childhood outcomes, which highlights how shifts in the policy can affect the hold of preventive interventions such as supplementation of folic acid.^[18]^ Access to prenatal imaging is also essential in diagnosis: the first ultrasound scan is typically performed between 8 and 12 weeks of gestation to confirm viability and estimate dating, while the second-trimester anomaly scan (18–22 weeks) is critical for detecting severe structural anomalies such as anencephaly. In underserved areas, lack of access to such imaging may delay diagnosis and decision-making.^[15,18]^

Landmark trials announced mandatory folic acid fortification in the United States which has reduced the incidence of neural tube defects (NTDs). The 1991 Medical Research Council randomized trial revealed that a supplementation of 4 mg folic acid daily showed a 72% reduction in NTDs.^[19]^ This was supported by a Hungarian randomized trial showing a 91% reduction in first-time NTDs with periconceptional multivitamin supplementation.^[20]^ These studies highlight the importance of folic acid prior to and during early pregnancy, especially in women of childbearing age. Thus, 0.4 mg folic acid was recommended daily for women of child bearing age by the U.S Public Health Service in 1992^[21]^ and by 1998, the FDA mandated folic acid fortification of cereal grains.^[22]^ As a result, the prevalence of anencephaly in the U.S declined from 4.2 to 2.1 per 10,000 live births between 1995 and 2006.^[23]^ However, serum folate levels dropped by 16% between 1999 and 2004, amongst women of reproductive age likely due to supplement usage and changes in dietary patterns.^[24]^

Disparities among NTD risks remain, especially pronounced amongst Hispanic women. Data from the Texas Birth Defects Registry show an anencephaly rate of 3.19 per 10,000 live births amongst Hispanics as compared to 2.17 for non-Hispanic Whites.^[25]^ Regarding cultural dietary notions, corn masa flour, unlike wheat- based products, is not necessarily fortified with folic acid due to FDA concerns of nutrient stability during its processing.^[26]^ Moreover, genetic variations like the MTHFR 677C > T polymorphism also plays a vital role. This mutation which reduces the efficiency of folate metabolism is homozygous in 32% of newborns in Mexico and has been associated with an increased risk of NTDs.^[27]^ Environmental exposures like the fumonisins from mold-contaminated corn, have also contributed to NTDs including the 1991 Brownsville, Texas outbreak which was well documented.^[28]^ These findings highlights that although significant public health benefits are seen with folic acid fortifications, further targeted strategies are necessary to reach at-risk populations.

The study presents a few limitations that should be considered. First, relying on ICD codes, as well as death certificate data, can result in underreporting or misclassification of anencephaly as a cause of death during the occurrence of multiple congenital anomalies or incomplete postnatal diagnosis. Another limitation may be that the datasets used do not contain detailed clinical information like prenatal diagnostic imaging, folic acid intake records, gestational age at diagnosis, or genetic data that might have helped refine the characterization of cases and enhanced etiologic insights. Third, their mortality datasets do not capture important contextual information, such as pregnancy termination decisions, when to intervene, and the quality and availability of prenatal care. In light of the existing disparities, these factors are exceedingly relevant for certain risk groups. Fourth, socioeconomic determinants of health substantially affect the incidence and outcomes of neural tube defects, like anencephaly, but are inconsistently available across surveillance systems. Finally, the inability to isolate single age groups on CDC WONDER, such as infants under 1 year of age, restricts the capacity to analyze age-specific trends and disparities, potentially overlooking critical insights.

## 5. Conclusion

Between 1999 and 2020, the United States saw a slow reduction in early childhood mortality caused by Anencephaly, wherein females showed a higher mortality rate compared to males throughout the study period. Hispanic/Latino individuals consistently bore the highest burden, and geographically, the highest mortality burden fell upon non-metropolitan areas, rates which remained consistently elevated throughout the period despite a significant overall decline. Sharp differences were also visible in the geographic picture. For example, the Midwestern and Southern states, particularly South Dakota, North Dakota, Oklahoma, and Arkansas, reported the highest AAMRs, almost 3 times higher than the rates in Northeastern states like Connecticut and Massachusetts. Targeted, community-specific interventions, particularly around folic acid access, prenatal education, and early screening, are essential to further reduce and equalize anencephaly-related mortality across regions.

## Author contributions

**Conceptualization:** Hashim Mohamed Siraj, Akshaya Srinivasan, Muhammad Husnain Ahmad, Angeline Abraham, Anushka Sen, Bilal Muhammad, Nikhila Liz Aby, Insha Samsulhuda Khan, Nirjhar Yaduvanshi, Mohammed Alsabri.

**Data curation:** Hashim Mohamed Siraj, Akshaya Srinivasan, Akshit Valsaraj Chemmancheri, Muhammad Husnain Ahmad, Angeline Abraham, Anushka Sen, Nikhila Liz Aby, Insha Samsulhuda Khan, Nirjhar Yaduvanshi, Mohammed Alsabri.

**Investigation:** Akshit Valsaraj Chemmancheri, Udgam Yaduvanshi.

**Methodology:** Akshit Valsaraj Chemmancheri, Nirjhar Yaduvanshi.

**Supervision:** Samiksha Ravindra Balaguragi, Nikhila Liz Aby.

**Validation:** Samiksha Ravindra Balaguragi, Muhammad Husnain Ahmad, Masab Ali, Ajin Mathews John, Nupura Ajesh, Nikhila Liz Aby, Muhammad Ibrahim, Courtney Truebody, Udgam Yaduvanshi, Mohammed Alsabri.

**Visualization:** Akshaya Srinivasan, Samiksha Ravindra Balaguragi, Muhammad Husnain Ahmad, Masab Ali, Ajin Mathews John, Bilal Muhammad, Nupura Ajesh, Muhammad Ibrahim, Courtney Truebody, Udgam Yaduvanshi, Mohammed Alsabri.

**Writing – original draft:** Hashim Mohamed Siraj, Vaishnavi Suresh, Muhammad Husnain Ahmad, Masab Ali, Angeline Abraham, Ajin Mathews John, Bilal Muhammad, Nupura Ajesh, Courtney Truebody.

**Writing – review & editing:** Hashim Mohamed Siraj, Vaishnavi Suresh, Muhammad Husnain Ahmad, Masab Ali, Angeline Abraham, Nupura Ajesh.

## Supplementary Material


