# Supplementary material for: Trends and disparities in anencephaly-related early childhood mortality in the United States between 1999 and 2020: A nationwide CDC WONDER analysis
Source: Medicine (Baltimore). 2025 Dec 26;104(52):e46593. doi: 10.1097/MD.0000000000046593 (PMC12746920; doi:10.1097/MD.0000000000046593)
Supplement: Supplementary file 1 [file medi-104-e46593-s001.docx]

**Supplemental Table 1** Anencephaly related Deaths, Stratified by Sex and Race in Early Childhood in United States, 1999 to 2020

| **DEATHS** | | | | | | | | | |
| --- | --- | --- | --- | --- | --- | --- | --- | --- | --- |
| **Year** | **Overall** | **Women** | **Men** | **NH White** | **NH Black or African American** | **NH Asian or Pacific Islander** | **NH American Indian or Alaska Native** | **Hispanic or Latino** | **Population** |
| 1999 | 326 | 196 | 130 | 264 | 43 | 15 | Missing | 87 | 19,135,544 |
| 2000 | 336 | 171 | 165 | 269 | 47 | 19 | Missing | 82 | 19,175,798 |
| 2001 | 283 | 159 | 124 | 233 | 38 | Missing | Missing | 83 | 19,298,217 |
| 2002 | 309 | 164 | 145 | 249 | 42 | 13 | Missing | 86 | 19,429,192 |
| 2003 | 348 | 186 | 162 | 292 | 42 | 12 | Missing | 113 | 19,592,446 |
| 2004 | 307 | 174 | 133 | 260 | 36 | Missing | Missing | 100 | 19,785,885 |
| 2005 | 329 | 173 | 156 | 266 | 47 | 11 | Missing | 98 | 19,917,400 |
| 2006 | 348 | 207 | 141 | 282 | 47 | 12 | Missing | 106 | 19,938,883 |
| 2007 | 336 | 177 | 159 | 272 | 50 | 11 | Missing | 113 | 20,125,962 |
| 2008 | 352 | 179 | 173 | 296 | 44 | Missing | Missing | 130 | 20,271,127 |
| 2009 | 335 | 166 | 169 | 279 | 39 | 12 | Missing | 105 | 20,244,518 |
| 2010 | 297 | 177 | 120 | 249 | 33 | 10 | Missing | 90 | 20,201,362 |
| 2011 | 286 | 157 | 129 | 235 | 42 | Missing | Missing | 89 | 20,162,058 |
| 2012 | 350 | 189 | 161 | 292 | 37 | 16 | Missing | 119 | 19,999,344 |
| 2013 | 311 | 179 | 132 | 270 | 32 | Missing | Missing | 81 | 19,868,088 |
| 2014 | 318 | 199 | 119 | 253 | 50 | 10 | Missing | 88 | 19,876,883 |
| 2015 | 304 | 173 | 131 | 252 | 41 | 10 | Missing | 96 | 19,907,281 |
| 2016 | 311 | 179 | 132 | 257 | 42 | 11 | Missing | 104 | 19,927,037 |
| 2017 | 333 | 187 | 146 | 282 | 36 | 11 | Missing | 106 | 19,938,860 |
| 2018 | 280 | 158 | 122 | 222 | 41 | 14 | Missing | 92 | 19,810,275 |
| 2019 | 289 | 162 | 127 | 242 | 32 | Missing | Missing | 75 | 19,576,683 |
| 2020 | 281 | 150 | 131 | 230 | 32 | 15 | Missing | 86 | 19,301,292 |
| **TOTAL** | 6969 | 3862 | 3107 | 5746 | 893 | 246 | 84 | 2129 | 435,484,135 |

NH - Non Hispanic

**Supplemental Table 2** Anencephaly–related Mortality, Stratified by Place of Death in Early Childhood in the United States, 1999 to 2020

| **DEATHS** | | | | | |
| --- | --- | --- | --- | --- | --- |
| **Year** | **Medical Facility** | **Home** | **Place of death unknown** | **Nursing home/ Long term Care Facility** | **Hospices** |
| 1999 | 304 | 16 | Missing | Missing | Missing |
| 2000 | 293 | 29 | Missing | Missing | Missing |
| 2001 | 250 | 21 | Missing | Missing | Missing |
| 2002 | 284 | 17 | Missing | Missing | Missing |
| 2003 | 312 | 24 | Missing | Missing | Missing |
| 2004 | 277 | 15 | Missing | Missing | Missing |
| 2005 | 295 | 28 | Missing | Missing | Missing |
| 2006 | 307 | 27 | Missing | Missing | Missing |
| 2007 | 306 | 17 | Missing | Missing | Missing |
| 2008 | 311 | 28 | Missing | Missing | Missing |
| 2009 | 290 | 27 | Missing | Missing | Missing |
| 2010 | 254 | 31 | Missing | Missing | Missing |
| 2011 | 257 | 22 | Missing | Missing | Missing |
| 2012 | 291 | 44 | Missing | Missing | Missing |
| 2013 | 272 | 26 | Missing | Missing | Missing |
| 2014 | 280 | 28 | Missing | Missing | Missing |
| 2015 | 274 | 21 | Missing | Missing | Missing |
| 2016 | 277 | 25 | Missing | Missing | Missing |
| 2017 | 304 | 21 | Missing | Missing | Missing |
| 2018 | 242 | 29 | Missing | Missing | Missing |
| 2019 | 250 | 27 | Missing | Missing | Missing |
| 2020 | 252 | 20 | Missing | Missing | Missing |
| **Total** | 6,182 | 543 | 10 | 21 | 41 |

**Supplemental Table 3:** Annual Percent Change (APC) of Anencephaly-Related Mortality per 100,000 in Early Childhood in the United States, 1999 to 2020

| **Annual Percentage Change (APC)** | | |
| --- | --- | --- |
| **Year Interval** | **APC (95% CI)** | |
| **Overall** | | |
| 1999-2020 | -0.43 (-0.94 to 0.09) | |
| **Male** | | |
| 1999-2008 | 0.30 (-2.55 to 3.23) | |
| 2008-2020 | -1.27 (-3.17 to 0.67) | |
| **Female** | | |
| 1999-2017 | 0.31 (-0.49 to 1.11) | |
| 2017-2020 | -5.42 (-16.93 to 7.68) | |
| **NH White** | | |
| 1999-2020 | -0.05 (-0.57 to 0.47) | |
| **NH Black or African American** | | |
| 1999-2020 | -1.20* (-2.08 to -0.31) | |
| **Hispanic or Latino** | | |
| 1999-2020 | -1.05* (-1.82 to -0.27) | |
| **NH Asian or Pacific Islander** | | |
| 1999-2005 | -10.87* (-19.95 to -0.74) | |
| 2005-2020 | -0.06 (-2.52 to 2.45) | |
| **Large Central Metro** | | |
| 1999-2017 | -0.48 (-1.26 to 0.30) | |
| 2017-2020 | -6.24 (-17.34 to 6.35) | |
| **Large Fringe Metro** | | |
| 1999-2015 | 1.25 (-0.57 to 3.10) | |
| 2015-2020 | -8.95 (-19.97 to 3.58) | |
| **Medium Metro** | | |
| 1999-2002 | 3.80 (-11.70 to 22.04) | |
| 2002-2020 | -0.37 (-1.25 to 0.52) | |
| **Small Metro** | | |
| 1999-2020 | -0.91 (-2.09 to 0.28) | |
| **Micropolitan** | | |
| 1999-2011 | -0.85 (-3.05 to 1.39) | |
| 2011-2020 | 3.33* (0.02 to 6.75) | |
| **NonCore** | | |
| 1999-2003 | | -4.93 (-20.59 to 13.80) |
| 2003-2020 | | 1.71 (-0.29 to 3.77) |

*Indicates that the annual percentage change (APC) is significantly different from zero at the alpha = 0.05 level. APC = annual percent change; NH = non-Hispanic.

**Supplemental Table 4** Overall and Sex‐Stratified Anencephaly–related Age-Adjusted Mortality Rates per 100,000 in Early Childhood in the United States, 1999 to 2020

| **Age-Adjusted Rate (95% CI)** | | | |
| --- | --- | --- | --- |
| **Year** | **Men** | **Women** | **Overall** |
| 1999 | 1.3 | 2.1 | 1.7 |
| 2000 | 1.7 | 1.8 | 1.8 |
| 2001 | 1.2 | 1.6 | 1.4 |
| 2002 | 1.4 | 1.7 | 1.6 |
| 2003 | 1.6 | 1.9 | 1.7 |
| 2004 | 1.3 | 1.8 | 1.5 |
| 2005 | 1.5 | 1.8 | 1.6 |
| 2006 | 1.4 | 2.1 | 1.7 |
| 2007 | 1.5 | 1.7 | 1.6 |
| 2008 | 1.6 | 1.7 | 1.7 |
| 2009 | 1.6 | 1.7 | 1.7 |
| 2010 | 1.2 | 1.8 | 1.5 |
| 2011 | 1.3 | 1.6 | 1.4 |
| 2012 | 1.6 | 1.9 | 1.8 |
| 2013 | 1.3 | 1.8 | 1.6 |
| 2014 | 1.2 | 2.1 | 1.6 |
| 2015 | 1.3 | 1.8 | 1.5 |
| 2016 | 1.3 | 1.9 | 1.5 |
| 2017 | 1.4 | 1.9 | 1.7 |
| 2018 | 1.2 | 1.7 | 1.4 |
| 2019 | 1.3 | 1.7 | 1.5 |
| 2020 | 1.4 | 1.6 | 1.5 |

**Supplemental Table 5** Anencephaly–related Age-Adjusted Mortality Rates per 100,000, Stratified by Race in Early Childhood in the United States, 1999 to 2020

| **Age-Adjusted Rate (95% CI)** | | | | | |
| --- | --- | --- | --- | --- | --- |
| **Year** | **NH White** | **NH Black or African American** | **NH American Indian or Alaska Native** | **Hispanic or Latino** | **NH Asian or Pacific Islander** |
| 1999 | 1.5 (1.3 - 1.7) | 1.4 (1.0 - 1.9) | Missing | 2.3 (1.8 - 2.8) | Unreliable  (0.1 - 0.2) |
| 2000 | 1.7 (1.4 - 1.9) | 1.5 (1.1 - 2.0) | Missing | 2.1 (1.7 - 2.6) | Unreliable  (0.1 - 0.2) |
| 2001 | 1.3 (1.1 - 1.5) | 1.1 (0.8 - 1.6) | Missing | 1.9 (1.5 - 2.3) | Missing |
| 2002 | 1.4 (1.2 - 1.6) | 1.3 (0.9 - 1.8) | Missing | 1.9 (1.6 - 2.4) | Unreliable  (0.1 - 0.2) |
| 2003 | 1.6 (1.3 - 1.8) | 1.4 (1.0 - 1.9) | Missing | 2.5 (2.0 - 2.9) | Unreliable  (0.0 - 0.1) |
| 2004 | 1.4 (1.2 - 1.6) | 1.2 (0.8 - 1.6) | Missing | 2.1 (1.7 - 2.5) | Missing |
| 2005 | 1.6 (1.3 - 1.8) | 1.4 (1.0 - 1.9) | Missing | 2.0 (1.6 - 2.4) | Unreliable  (0.0 - 0.1) |
| 2006 | 1.6 (1.4 - 1.9) | 1.4 (1.0 - 1.9) | Missing | 2.1 (1.7 - 2.5) | Unreliable  (0.0 - 0.1) |
| 2007 | 1.4 (1.2 - 1.7) | 1.5 (1.1 - 2.0) | Missing | 2.1 (1.7 - 2.5) | Unreliable  (0.0 - 0.1) |
| 2008 | 1.5 (1.3 - 1.7) | 1.4 (1.0 - 1.9) | Missing | 2.4 (2.0 - 2.8) | Missing |
| 2009 | 1.7 (1.4 - 1.9) | 1.1 (0.8 - 1.5) | Missing | 2.0 (1.6 - 2.4) | Unreliable  (0.0 - 0.1) |
| 2010 | 1.6 (1.3 - 1.8) | 1.0 (0.7 - 1.4) | Missing | 1.8 (1.4 - 2.2) | Unreliable  (0.0 - 0.1) |
| 2011 | 1.4 (1.2 - 1.6) | 1.3 (0.9 - 1.8) | Missing | 1.7 (1.4 - 2.1) | Missing |
| 2012 | 1.7 (1.4 - 1.9) | 1.2 (0.8 - 1.6) | Missing | 2.3 (1.9 - 2.7) | Unreliable  (0.1 - 0.2) |
| 2013 | 1.9 (1.6 - 2.1) | 0.9 (0.6 - 1.3) | Missing | 1.6 (1.3 - 2.0) | Missing |
| 2014 | 1.6 (1.4 - 1.9) | 1.4 (1.0 - 1.9) | Missing | 1.7 (1.4 - 2.1) | Unreliable  (0.0 - 0.1) |
| 2015 | 1.5 (1.3 - 1.8) | 1.2 (0.8 - 1.7) | Missing | 1.9 (1.5 - 2.3) | Unreliable  (0.0 - 0.1) |
| 2016 | 1.5 (1.2 - 1.7) | 1.1 (0.8 - 1.6) | Missing | 2.0 (1.6 - 2.4) | Unreliable  (0.0 - 0.1) |
| 2017 | 1.7 (1.5 - 2.0) | 1.1 (0.8 - 1.6) | Missing | 2.0 (1.6 - 2.4) | Unreliable  (0.0 - 0.1) |
| 2018 | 1.3 (1.1 - 1.6) | 1.3 (0.9 - 1.7) | Missing | 1.8 (1.4 - 2.2) | Unreliable  (0.0 - 0.1) |
| 2019 | 1.7 (1.5 - 2.0) | 1.1 (0.8 - 1.6) | Missing | 1.5 (1.2 - 1.9) | Missing |
| 2020 | 1.6 (1.3 - 1.8) | 0.8 (0.5 - 1.2) | Missing | 1.8 (1.4 - 2.2) | Unreliable  (0.0 - 0.1) |

**Supplemental Table 6** Anencephaly–related Age-Adjusted Mortality Rates per 100,000, Stratified by States in Early Childhood in the United States, 1999 to 2020

| **State** | **Age-Adjusted Rate (95% CI)** |
| --- | --- |
| **Alabama** | 1.9 (1.5 - 2.2) |
| **Alaska** | Unreliable (0.9 - 2.5) |
| **Arizona** | 2.0 (1.7 - 2.2) |
| **Arkansas** | 2.4 (2.0 - 2.9) |
| **California** | 1.4 (1.3 - 1.5) |
| **Colorado** | 1.2 (1.0 - 1.5) |
| **Connecticut** | 0.5 (0.3 - 0.8) |
| **Delaware** | 1.9 (1.2 - 2.8) |
| **District of Columbia** | Unreliable (0.7 - 2.3) |
| **Florida** | 1.5 (1.3 - 1.6) |
| **Georgia** | 1.4 (1.2 - 1.6) |
| **Hawaii** | 1.4 (1.0 - 2.1) |
| **Idaho** | 1.6 (1.1 - 2.2) |
| **Illinois** | 1.5 (1.3 - 1.6) |
| **Indiana** | 2.4 (2.1 - 2.7) |
| **Iowa** | 1.6 (1.3 - 2.0) |
| **Kansas** | 2.8 (2.3 - 3.3) |
| **Kentucky** | 1.6 (1.3 - 1.9) |
| **Louisiana** | 2.1 (1.8 - 2.5) |
| **Maine** | 2.1 (1.4 - 2.9) |
| **Maryland** | 1.4 (1.1 - 1.6) |
| **Massachusetts** | 0.8 (0.6 - 1.0) |
| **Michigan** | 1.9 (1.6 - 2.1) |
| **Minnesota** | 1.8 (1.5 - 2.1) |
| **Mississippi** | 1.8 (1.4 - 2.3) |
| **Missouri** | 2.4 (2.0 - 2.7) |
| **Montana** | Unreliable (0.8 - 2.2) |
| **Nebraska** | 2.4 (1.8 - 3.0) |
| **Nevada** | 1.2 (0.8 - 1.6) |
| **New Hampshire** | Unreliable (0.6 - 1.7) |
| **New Jersey** | 0.8 (0.6 - 1.0) |
| **New Mexico** | 1.1 (0.8 - 1.6) |
| **New York** | 0.8 (0.7 - 0.9) |
| **North Carolina** | 1.6 (1.4 - 1.8) |
| **North Dakota** | 3.0 (2.0 - 4.2) |
| **Ohio** | 1.8 (1.6 - 2.1) |
| **Oklahoma** | 2.4 (2.0 - 2.8) |
| **Oregon** | 1.4 (1.1 - 1.7) |
| **Pennsylvania** | 1.6 (1.4 - 1.8) |
| **Rhode Island** | Unreliable (0.6 - 1.8) |
| **South Carolina** | 1.4 (1.1 - 1.7) |
| **South Dakota** | 3.5 (2.5 - 4.7) |
| **Tennessee** | 1.8 (1.5 - 2.1) |
| **Texas** | 2.0 (1.9 - 2.1) |
| **Utah** | 1.8 (1.5 - 2.2) |
| **Virginia** | 1.6 (1.4 - 1.8) |
| **Washington** | 1.3 (1.1 - 1.6) |
| **West Virginia** | 1.8 (1.3 - 2.4) |
| **Wisconsin** | 1.7 (1.4 - 2.0) |
| **Wyoming** | Unreliable (0.6 - 2.4) |

**Supplemental Table 7** Anencephaly–related Age-Adjusted Mortality Rates per 100,000, Stratified by Census Region in Early Childhood in the United States, 1999 to 2020

| **Census Region** | **Year** | **Age-Adjusted Rate (95% CI)** |
| --- | --- | --- |
| **Northeast** | 1999 | 1.3 (0.9 - 1.7) |
| **Northeast** | 2000 | 1.4 (1.0 - 1.8) |
| **Northeast** | 2001 | 0.9 (0.6 - 1.3) |
| **Northeast** | 2002 | 0.9 (0.6 - 1.2) |
| **Northeast** | 2003 | 1.0 (0.7 - 1.4) |
| **Northeast** | 2004 | 0.9 (0.6 - 1.3) |
| **Northeast** | 2005 | 1.1 (0.8 - 1.6) |
| **Northeast** | 2006 | 1.0 (0.7 - 1.4) |
| **Northeast** | 2007 | 0.8 (0.6 - 1.2) |
| **Northeast** | 2008 | 1.2 (0.9 - 1.7) |
| **Northeast** | 2009 | 1.0 (0.7 - 1.4) |
| **Northeast** | 2010 | 0.9 (0.6 - 1.3) |
| **Northeast** | 2011 | Unreliable (0.3 - 0.8) |
| **Northeast** | 2012 | 1.0 (0.7 - 1.4) |
| **Northeast** | 2013 | 1.2 (0.8 - 1.6) |
| **Northeast** | 2014 | 0.8 (0.5 - 1.2) |
| **Northeast** | 2015 | 0.9 (0.6 - 1.3) |
| **Northeast** | 2016 | 1.0 (0.7 - 1.4) |
| **Northeast** | 2017 | 1.1 (0.8 - 1.6) |
| **Northeast** | 2018 | 1.1 (0.8 - 1.6) |
| **Northeast** | 2019 | 0.7 (0.5 - 1.1) |
| **Northeast** | 2020 | 0.8 (0.5 - 1.2) |
| **Northeast** | **Total** | 1.0  (0.9 - 1.1) |
| **Midwest** | 1999 | 1.9  (1.5 - 2.4) |
| **Midwest** | 2000 | 2.2  (1.8 - 2.7) |
| **Midwest** | 2001 | 1.4  (1.1 - 1.8) |
| **Midwest** | 2002 | 2.2  (1.7 - 2.6) |
| **Midwest** | 2003 | 2.0  (1.6 - 2.5) |
| **Midwest** | 2004 | 1.7  (1.3 - 2.1) |
| **Midwest** | 2005 | 2.2  (1.8 - 2.7) |
| **Midwest** | 2006 | 2.5  (2.0 - 3.0) |
| **Midwest** | 2007 | 1.6  (1.2 - 2.0) |
| **Midwest** | 2008 | 2.1  (1.7 - 2.5) |
| **Midwest** | 2009 | 2.0  (1.6 - 2.5) |
| **Midwest** | 2010 | 1.6  (1.3 - 2.1) |
| **Midwest** | 2011 | 1.9  (1.5 - 2.3) |
| **Midwest** | 2012 | 2.1  (1.7 - 2.6) |
| **Midwest** | 2013 | 2.1  (1.7 - 2.6) |
| **Midwest** | 2014 | 2.0  (1.6 - 2.5) |
| **Midwest** | 2015 | 2.1  (1.7 - 2.6) |
| **Midwest** | 2016 | 1.9  (1.5 - 2.4) |
| **Midwest** | 2017 | 2.1  (1.7 - 2.6) |
| **Midwest** | 2018 | 1.5  (1.1 - 1.9) |
| **Midwest** | 2019 | 1.8  (1.4 - 2.3) |
| **Midwest** | 2020 | 1.7  (1.4 - 2.2) |
| **Midwest** | **Total** | 1.9  (1.8 - 2.0) |
| **South** | 1999 | 1.7  (1.4 - 2.0) |
| **South** | 2000 | 1.7  (1.4 - 2.1) |
| **South** | 2001 | 1.7  (1.4 - 2.0) |
| **South** | 2002 | 1.5  (1.2 - 1.8) |
| **South** | 2003 | 2.0  (1.6 - 2.3) |
| **South** | 2004 | 1.7  (1.4 - 2.0) |
| **South** | 2005 | 1.7  (1.4 - 2.0) |
| **South** | 2006 | 1.7  (1.4 - 2.0) |
| **South** | 2007 | 1.9  (1.6 - 2.2) |
| **South** | 2008 | 1.6  (1.3 - 1.9) |
| **South** | 2009 | 1.8  (1.5 - 2.1) |
| **South** | 2010 | 1.8  (1.5 - 2.1) |
| **South** | 2011 | 1.7  (1.4 - 2.0) |
| **South** | 2012 | 2.0  (1.7 - 2.3) |
| **South** | 2013 | 1.5  (1.3 - 1.8) |
| **South** | 2014 | 2.0  (1.7 - 2.3) |
| **South** | 2015 | 1.6  (1.3 - 1.8) |
| **South** | 2016 | 1.9  (1.6 - 2.2) |
| **South** | 2017 | 1.8  (1.5 - 2.1) |
| **South** | 2018 | 1.6  (1.3 - 1.9) |
| **South** | 2019 | 1.8  (1.5 - 2.1) |
| **South** | 2020 | 1.9  (1.5 - 2.2) |
| **South** | **Total** | 1.7  (1.7 - 1.8) |
| **West** | 1999 | 1.7  (1.4 - 2.2) |
| **West** | 2000 | 1.6  (1.3 - 2.0) |
| **West** | 2001 | 1.3  (1.0 - 1.7) |
| **West** | 2002 | 1.6  (1.2 - 2.0) |
| **West** | 2003 | 1.7  (1.4 - 2.2) |
| **West** | 2004 | 1.5  (1.2 - 1.9) |
| **West** | 2005 | 1.3  (1.0 - 1.7) |
| **West** | 2006 | 1.5  (1.2 - 1.9) |
| **West** | 2007 | 1.7  (1.4 - 2.1) |
| **West** | 2008 | 1.8  (1.4 - 2.2) |
| **West** | 2009 | 1.6  (1.3 - 2.0) |
| **West** | 2010 | 1.4  (1.1 - 1.8) |
| **West** | 2011 | 1.3  (1.0 - 1.6) |
| **West** | 2012 | 1.7  (1.3 - 2.1) |
| **West** | 2013 | 1.5  (1.2 - 1.9) |
| **West** | 2014 | 1.2  (0.9 - 1.6) |
| **West** | 2015 | 1.4  (1.1 - 1.8) |
| **West** | 2016 | 1.1  (0.8 - 1.4) |
| **West** | 2017 | 1.5  (1.2 - 1.9) |
| **West** | 2018 | 1.4  (1.0 - 1.7) |
| **West** | 2019 | 1.3  (1.0 - 1.7) |
| **West** | 2020 | 1.1  (0.8 - 1.5) |
| **West** | **Total** | 1.5  (1.4 - 1.5) |
| **Total** | **Total** | 1.6  (1.6 - 1.6) |

**Supplemental Table 8** Anencephaly-related Age-Adjusted Mortality Rates per 100,000, Stratified by Urban-Rural Classification in Early Childhood in the United States, 1999 to 2020

| **Year** | **Metropolitan** | **Nonmetropolitan** |
| --- | --- | --- |
| 1999 | 270 | 56 |
| 2000 | 273 | 63 |
| 2001 | 227 | 56 |
| 2002 | 256 | 53 |
| 2003 | 296 | 52 |
| 2004 | 269 | 38 |
| 2005 | 270 | 59 |
| 2006 | 290 | 58 |
| 2007 | 283 | 53 |
| 2008 | 287 | 65 |
| 2009 | 283 | 52 |
| 2010 | 240 | 57 |
| 2011 | 234 | 52 |
| 2012 | 284 | 66 |
| 2013 | 251 | 60 |
| 2014 | 269 | 49 |
| 2015 | 255 | 49 |
| 2016 | 258 | 53 |
| 2017 | 273 | 60 |
| 2018 | 227 | 53 |
| 2019 | 220 | 69 |
| 2020 | 228 | 53 |


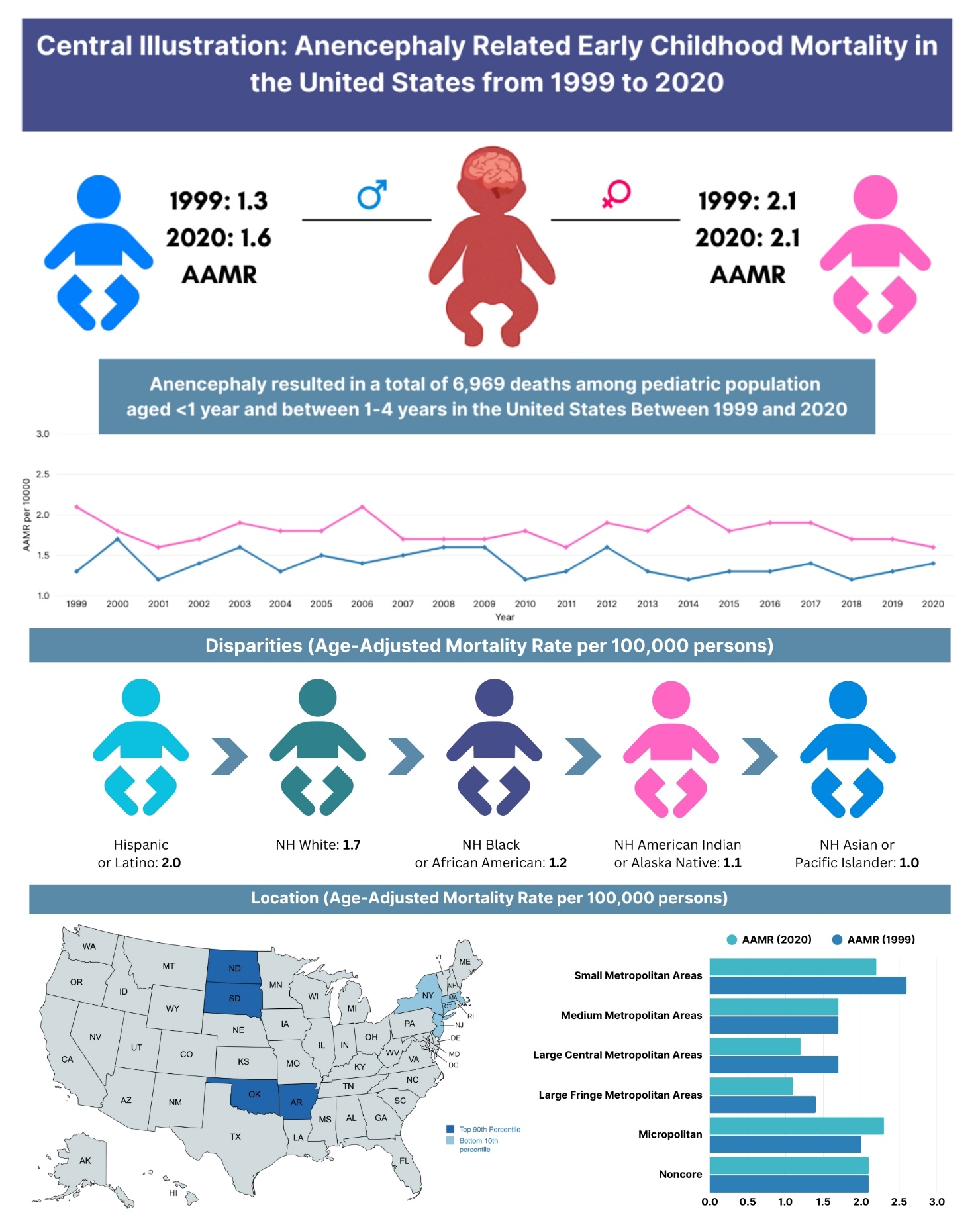


Central Illustration: Trends and Disparities in Anencephaly-Related Early Childhood Mortality in the United States between 1999 and 2020: A Nationwide Analysis
